# Chasing ‘vulnerability’ across six decades of the Declaration of Helsinki

**DOI:** 10.1007/s40592-025-00235-4

**Published:** 2025-02-25

**Authors:** Oskar Lindholm, Sakari Karjalainen, Veikko Launis

**Affiliations:** 1 University of Turku, Turku, Finland; 2 Tampere University, Tampere, Finland

**Keywords:** Research ethics, Vulnerability, Vulnerable populations, The Declaration of Helsinki, Research Policy, Informed Consent

## Abstract

The year 2024 marked the 60th anniversary of the World Medical Association’s Declaration of Helsinki (DoH). Coincidentally, the WMA published the 8th revision of this landmark document guiding medical research involving human subjects. One of the key changes in this latest revision concerns the notion of vulnerability, which has always been central to the DoH’s ethos. The term ‘vulnerability’ was explicitly introduced in the 5th revision, published in 2000, which lists five vulnerable groups. Subsequent revisions have significantly altered how vulnerability is portrayed and understood within the document. This article traces the conceptualisation of vulnerability across the various versions of the DoH, culminating in its recently published 8th revision. We explore the underlying principles of each revision and examine how these principles have both influenced and been influenced by broader ethical discourses. Lastly, we address some of the challenges that future revisions must meet to ensure that the document remains internally coherent and practically applicable for researchers and research ethics committees alike.

## Introduction

The year 2024 marked the 60th anniversary of the World Medical Association’s (WMA’s) Declaration of Helsinki (DoH) and the publication of its 8th and latest revision (WMA 2024a). Since its first publication in 1964, the DoH has undergone eight revisions and become a landmark document guiding medical research involving human subjects. As of today, it serves as a guiding framework for physicians, ethics review committees, research funding organisations, researchers, and research participants, and is frequently used to assess the ethical standards of clinical trials (Millum et al. 2013). Other documents and guidelines, such as those of the Council for International Organizations of Medical Sciences (CIOMS), the International Conference on Harmonization – Good Clinical Practice (ICH-GCP) guidelines, and the UNESCO Declaration on Bioethics and Human Rights, explicitly refer to the DoH in their respective texts (CIOMS 2016; ICH 2016; UNESCO 2005). The DoH has also been both directly and indirectly influential in shaping national and transnational legislation (Goodyear et al. 2007). An example of this influence is the EU Clinical Trials Regulation, effective since January 2022, which specifically refers to the ethical principles of the DoH (European Parliament and European Council 2014).

While concern for human vulnerability has always been central to the DoH’s ethos, the term ‘vulnerability’ was explicitly introduced only in the 5th revision, published in 2000 (WMA 2000). In this revision, vulnerability is highlighted in paragraph 8 of the introduction, according to which ‘some research populations are vulnerable and need special protection’. Five examples of vulnerable groups are mentioned. In the 6th revision in 2008, vulnerability is no longer presented in the introduction but rather under the list of principles (WMA 2008). Two criteria for vulnerability that correspond to two of the groups mentioned in the 5th revision are mentioned: undue influence and coercion. In the 7th revision in 2013, vulnerability is addressed in a wholly separate section on ‘Vulnerable Groups and Individuals’ (WMA 2013). No examples of vulnerable groups are given. Rather, paragraph 19 of the 7th revision connects vulnerability with ‘an increased likelihood of being wronged or of incurring additional harm’.

The 8th and latest revision of the DoH, published in October 2024, retains the wrongs-based formulation of vulnerability and further emphasises the context-dependent and dynamic nature of vulnerability (WMA 2024a). The extended paragraph 19 no longer refers to vulnerable individuals per se; rather, it states that some individuals, groups, and communities may find themselves in ‘a situation of more vulnerability as research participants’ due to factors that may be fixed, contextual, or dynamic. Furthermore, the 8th revision highlights that exclusion from medical research may perpetuate or exacerbate (health) disparities. Therefore, the harms of exclusion must be carefully considered and weighed against the potential harms of inclusion.

The changes in the DoH’s statements on vulnerability reflect broader conceptual advancements in the understanding of vulnerability as an independent principle in bioethics. Especially in recent years, a growing body of literature has worked to refine the way in which vulnerability is conceptualised and applied (Armstrong 2017; Boldt 2019; Bracken-Roche et al. 2017; Hurst 2008; Luna 2009; Luna 2019; Martin et al. 2014; Rogers et al. 2012; ten Have 2015; ten Have 2016). An important insight from this body of work is that vulnerability is a fundamental ethical principle, meaning that it cannot be reduced to issues concerning autonomous, free and informed consent (IBC 2013; ten Have 2016). Additionally, vulnerability does not necessarily arise from (supposed) internal properties of research subjects but can stem from aspects of the research itself (Luna 2009; Levine et al. 2004). This broader understanding of vulnerability raises ethical considerations that include not only the need for protection but also questions of justice, solidarity, and responsibility (ten Have 2015).

This article traces the conceptualisation of vulnerability across the various versions of the DoH, culminating in the recently published 8th revision. We examine the underlying principles of each revision and how these principles have both shaped and been shaped by broader ethical discourses. Lastly, we address some of the challenges that future revisions must meet to ensure that the document remains internally coherent and practically applicable for researchers and research ethics committees alike.

## Vulnerability

Clinical research is a significant ethical challenge. The ultimate goal is to develop generalisable and applicable knowledge for the benefit of future patients; research participants serve as the means for achieving this objective. Robust ethical standards are required to ensure that participants are not treated merely as means to an end while contributing to the common good. Specifically, individuals may participate as subjects in experiments only if certain conditions are met and certain safeguards are in place. These include, among others, a reasonable risk–benefit ratio, minimisation of risks, voluntary informed consent, and fair recruitment of subjects. (Emanuel et al. 2000).

In this context, vulnerability emerges as a central concept for bioethical discourse. Foundational documents such as the DoH and the Nuremberg Code are grounded in the recognition that individuals might suffer harm while participating in research (Rogers et al. 2012). In other words, research participants are often *vulnerable* while participating in research.^1^ Principles such as informed consent and a reasonable risk–benefit ratio are employed to protect both the rights and welfare of those who participate (Emanuel et al. 2000). In the 8th and latest revision of the DoH, this universal ‘vulnerability concern’ is articulated in the General Principles section, which formulates why research ethics ultimately is needed (Carlson et al. 2004; WMA 2024a). The DoH states that medical research must involve research on human subjects (paragraph 5). While it would be unethical to refrain from challenging current methods in medical practice through research, individuals cannot be treated merely as means to an end (paragraph 7). Ultimately, it is the duty of the physician to protect the life, well-being, dignity, and rights of all research participants (paragraphs 4, 6, and 9).

In the literature, vulnerability is commonly thought of as an either universal or circumstantial phenomenon (Armstrong 2017). Universal (or ontological) approaches to vulnerability consider vulnerability an inescapable and inherent aspect of human existence (ibid.; Hoffmaster 2006; Rendtorff 2002; Rendtorff and Kemp 2000). Every human being is always vulnerable to some degree; hence the agreement on equal protection for all (Kottow 2003).^2^ In contrast, circumstantial (or situational) approaches to vulnerability focus on vulnerability that for some reason is ‘greater than ordinary’ (Rogers et al. 2012, p. 12). Circumstantial approaches to vulnerability are of particular importance in research ethics and regulatory guidelines, where vulnerability is often understood as a risk of harm or wrong that exceeds that faced by ‘paradigmatic’ subjects in similar circumstances (Bracken-Roche et al. 2017). From a regulatory perspective vulnerability can be thought of as highlighting instances where the conditions for ethical research – the underlying ‘deal’ that underlies human subjects research and is expressed in various guidelines – are at increased risk of being compromised (Coleman 2009). Vulnerability thus implies a need for special protection and prompts researchers and ethics boards to implement specific measures to safeguard those affected (Hurst 2008). While equal protection suffices for ‘ordinary’ vulnerability, ‘special’ or ‘particular’ vulnerability that arises due to situational factors most often requires tailored measures to protect those subjects affected (Martin et al. 2014).

The first guideline to mention vulnerability was the 1978 US Belmont Report (National Commission for the Protection of Human Subjects of Biomedical and Behavioral Research 1978). Since then, a growing number of legal and non-legal guidelines and ethics policies have addressed vulnerability, reflecting an expanded understanding of both its scope and its normative force (Bracken-Roche et al. 2017). Ten Have (2015, p. 395) describes the emergence of vulnerability as an independent ethical principle as follows:

Initially vulnerability was considered to be a special consideration in the application of the general principles of respect for persons, beneficence, and justice, relevant in the context of research with human beings. The 1991 Council for International Organizations of Medical Sciences (CIOMS) guidelines were the first to refer to vulnerability as a (secondary) principle incorporated in the principle of respect for persons. Later, in the 1993 CIOMS guidelines, the concept of ‘vulnerability’ evolved to include a special application of the principle of respect for persons and the principle of justice, considered as a guideline for research itself. Vulnerability is promoted in the UNESCO Declaration [The Universal Declaration on Bioethics and Human Rights] as a fundamental bioethical principle – no longer only relevant for medical research but also for healthcare. The sixth revision of the Declaration of Helsinki (World Medical Association [WMA] 2008) also classifies vulnerability in the category of principles.

Vulnerability has nevertheless proven to be difficult to conceptualise and employ in research ethics. Common definitions of vulnerability have been criticised as either too broad or too narrow to be practically useful (Hurst 2008; Levine et al. 2004). In particular, universal approaches to vulnerability are often considered too broad and vague to function as a basis upon which the special protection associated with circumstantial vulnerability can be formed.^3^ Conversely, some accounts of circumstantial vulnerability employed in research ethics have focused exclusively on some specific traits or circumstances – a lack of capacity to consent, for example – and have thus excluded individuals that for other reasons might be at risk in a research setting.

In research ethics, vulnerability has traditionally been understood through a ‘subpopulation approach’ (Luna 2019, p. 88), meaning that the locus of vulnerability has typically been a group rather than an individual.^4^ Due to vulnerability being historically framed primarily in terms of autonomous informed consent, conventional groups of vulnerable individuals are children, prisoners, and persons with mental or behavioural disorders (CIOMS 2002; ten Have 2016). Other conventional vulnerable groups include, for example, junior or subordinate members of hierarchical groups, elderly persons, residents of nursing homes, poor people, patients with incurable diseases, and refugees (CIOMS 2002).

While the topic and category of vulnerable populations or groups has a long-standing tradition in medical and epidemiological literature, contemporary bioethics increasingly emphasises approaches that are deemed more nuanced and individualised (Macioce 2022).^5^ A key critique of the subpopulation approach is that it stereotypes and labels entire groups without recognising individual differences within those groups (Levine et al. 2004; Luna 2009). Further, certain groups traditionally identified as vulnerable – such as children, pregnant women, and the elderly – have historically been excluded from research on grounds of safety and protection (Chambers et al. 2008; Langmann 2023; Rogers et al. 2012). Arguably, such exclusionary practices have led to a shortage of appropriate treatments and medications for individuals in these groups, and their increased vulnerability (Blehar et al. 2013; Macklin 2010; van der Zande et al. 2017). Perhaps reflecting this criticism, research guidelines and ethical documents have generally shortened their lists of vulnerable groups or stopped identifying groups altogether.

In research ethics, vulnerability remains tightly linked to protection, although the specific nature of this protection is seldom elaborated further (Zagorac 2016). Ten Have (2016) identifies two other major responses to vulnerability in research ethics guidelines: extra justification and the sharing of benefits. He considers extra justification the oldest response to vulnerability, and traces it as far back as the Belmont report of 1978 (ibid.). The rationale in the Belmont Report was that some populations were more likely to bear the burdens of research, and could only be subjects in research if an adequate justification for their inclusion could be given (Beauchamp 2008). In the 1990s, growing concerns about the consequences of excluding certain populations from research altogether led to the idea of vulnerability implying specifically considered protection (ten Have 2016). By employing special safeguards those deemed vulnerable would be able to participate in research safely, and the problems linked to exclusion would diminish. However, as vulnerability was still framed primarily as a problem of individual decision-making, the ‘protection’ often entailed exclusion in practice, as noted by ten Have (ibid.).

The most recent and broadest response to vulnerability, namely the sharing of benefits, draws on universal approaches to vulnerability and sees vulnerability as generating positive duties for researchers and ethics committees (ibid.). From this perspective, our shared vulnerability ‘calls on every human being, especially those who have the responsibility to advance knowledge and to decide how to use it, to fulfil the fundamental obligations we have one to another.’ (IBC 2013, p.13) In this view, vulnerability is linked not only to decision-making or autonomy but also to concepts such as integrity, dignity, and solidarity (ibid). Sharing of benefits entails that research on vulnerable populations needs to be responsive to the broader needs of the populations researched on and should seek to address health issues of these populations (or populations in similar situations). Further, participants should have reasonable access to interventions identified as beneficial by the research after the study has ended (WMA 2024a, paragraph 34). While vulnerability may sometimes entail protection of certain groups or individuals, vulnerability also entails a sense of shared responsibility and a commitment to justice on a broad scale.

To summarise, then, vulnerability is typically understood as a susceptibility to suffer harm or wrong in research ethics.^6^ Some individuals are more vulnerable than others, meaning they have an increased likelihood of suffering said harm or wrong. This heightened susceptibility is typically referred to as ‘special’ or ‘particular’ vulnerability and obligates various stakeholders in research to go beyond the baseline protections or responses provided in general guidelines or regulations. Importantly, the response to vulnerability should consider the contextual factors that produce, maintain, or exacerbate vulnerability: group characterisation based exclusively on some supposed ‘internal’ properties is widely considered a crude and potentially harmful way of employing vulnerability (Levine et al. 2004; Luna 2009). Exclusion may lead to additional harm or wrong, underscoring the need for more detailed and nuanced approaches to addressing vulnerability.

## The Declaration of Helsinki

The DoH is the first and most influential international set of ethical principles that guide medical research involving human subjects (Ehni and Wiesing 2024).^7^ Its roots lie in the Nuremberg Code of 1947, which was written as part of the so-called Doctor’s Trial as a reaction to the atrocities committed by Nazi physicians and investigators during WWII (Carlson et al. 2004). The Nuremberg Code in turn derived in part from a 1931 German government set of guidelines for therapeutic and scientific research on human subjects (Vollman and Winau 1996; Ghooi 2011).

The original DoH was introduced in 1964 to address perceived shortcomings in the Nuremberg Code, particularly concerning physicians conducting research on patients (Emanuel et al. 2000). As noted, its influence on national and international legislation, regulations, guidelines, and other non-binding documents has been vast to the extent that any fair account is a formidable task.^8^ The DoH has been described as ‘the cornerstone of biomedical research’ and ‘the largely unquestioned anchor for ethical decision-making in clinical trials’ (Crawley and Hoet 1999).^9^ Beyleveld and Brownsword (2001, p. 29) go as far as to say that ‘[m]odern bioethics has its origins in the Code of Nuremberg of 1947 and gathers pace with the Declaration of Helsinki in 1964.’ It might also be noted that journal publishers often require that biomedical research published in their respective journals comply with the DoH, a requirement included in paragraph 36 of the 8th and latest revision (WMA 2024a). While originally addressed primarily to physicians, the document has long served as a framework for both physicians, journal publishers, ethics review committees, research funding organisations, researchers, and research participants alike (Millum et al. 2013). This broader applicability is reflected in the updated paragraph 2 of the 8th and latest revision, which states that the principles in the DoH ‘should be upheld by all individuals, teams, and organizations involved in medical research, as [they] are fundamental to respect for and protection of all research participants, including both patients and healthy volunteers.’ Furthermore, many paragraphs in the latest revision now refer to ‘physicians and other researchers’, acknowledging the interdisciplinary nature of modern medical research (Resneck 2025).^10^

The DoH has undergone eight revisions and been amended with two notes of clarifications since 1964. The revisions have mainly been additive in nature, leading to the latest 8th revision being almost four times the size of the DoH of 1964. In 1964, the DoH comprised 11 articles and 713 words, while its latest revision stands at 37 articles and 2769 words.^11^ Between these versions, the structure of the document has changed significantly. Among the revisions, those in 1975, 2000, and 2013 stand out for their extensive changes in structure and/or content. Prior to the 5th revision in 2000, the document followed a similar pattern where an introductory section was followed by numbered paragraphs under three headings: ‘Basic Principles’, ‘Medical Research Combined with Professional Care’, and ‘Non-therapeutic Biomedical Research Involving Human Subjects’ (Carlson et al. 2004). This structure reflected a dichotomy between ‘therapeutic’ and ‘non-therapeutic’ research endorsed by the 1964 DoH (WMA 1964). This dichotomy was abandoned in the 5th revision, leading the way for a new structure that persisted through the 6th revision. In the 5th revision, the introduction featured numbered paragraphs detailing both some principles and the overall framework of the document (Carlson et al. 2004). The introduction was then followed by sections headed ‘Basic Principles for All Medical Research’ and ‘Additional Principles for Medical Research Combined with Medical Care’. The 5th revision thus represented a ‘major logical re-framing’ of how the DoH approached research on human subjects (ibid., p. 699).

The current structure was adopted in the 7th revision in 2013. Both the 7th and 8th revision use a subheading system where paragraphs concerning the same topic are grouped together (WMA 2013; WMA 2024a). In the 8th revision, for example, paragraphs 3–15 concern ‘General Principles’; paragraphs 19–20 ‘Individual, Group, and Community Vulnerability’; and paragraphs 25–32 ‘Free and Informed Consent’. This structure has been praised for its readability, logical organisation, and precision, in addition to being deemed more effective at protecting research participants (Millum et al. 2013; Muthuswamy 2014; Ehni and Wiesing 2024). Other subheading titles include, for example, ‘Risks, Burdens, and Benefits’, ‘Research Ethics Committees’, ‘Privacy and Confidentiality’, and ‘Use of Placebo’ (WMA 2024a).

The frequency with and extent to which the DoH should be revised remains a matter of debate. Emanuel (2013, p. 1533) suggests that the DoH should strive for ‘tentative immortality’, meaning that it should ‘establish universal, minimum standards without which research is unethical’; the interpretation and application of the principles in the DoH should be made elsewhere. Others see regular revisions as necessary for keeping in touch with the evolving field of medical research (Ehni and Wiesing 2024). As a general rule, the DoH has grown progressively more prescriptive over time, presenting not only universal principles but also detailed specifications of said principles (Goodyear et al. 2007). The increased prescriptiveness might be viewed as part of a general trend towards *legalisation* in bioethics, defined by Abbott et al. (2000) as a set of characteristics with three dimensions: obligation, precision, and delegation. From this perspective, the WMA serves as a legislator, providing the precise and ‘steering’ DoH; the REC’s as delegated arbitrators; and researchers as obligated subjects (Johnsson et al. 2014). In short, the trend increasingly seems to be toward viewing (bio)ethics as ‘the application of ready-made concepts and rules’ (Eriksson 2010, as quoted in Johnsson et al. 2014), and ethical guidelines as checklists that clearly delineate what researchers should and should not do in different situations.^12^ One factor contributing to this development could be a ‘perceived necessity for accountability’ within an environment of ‘institutionalized distrust’ (Johnsson et al. 2014). From this perspective, the DoH would ideally contain precise and highly elaborated rules as opposed to vague, general principles, and these rules would be uniformly agreed upon by all parties involved (ibid.). However, it is not clear that the increased specificity of the content in the DoH diminishes the need for interpretation and potential dissent (Goodyear et al. 2007). In a changing medical landscape, highly elaborated rules arguably make further revisions more likely and more necessary than universal principles without which research can be considered unethical (ibid.). At times, stakeholders have chosen to follow earlier revisions of the DoH so as to avoid specific provisions introduced in more recent revisions (Ndebele 2013; Wolinsky 2006).

The DoH can be conceived as a ‘living document’ shaped by both continuous public discourse and changing circumstances (Carlson et al. 2004, p. 696). The 1st revision explicitly states: ‘[the recommendations] should be kept under review in the future’ (WMA 1975). Importantly, the environment around the DoH importantly includes not only the technical and scientific developments in medical research but also the readers’ interpretations and responses to the DoH, alongside the emergence of competing guidelines (Carlson et al. 2004). While the DoH was unique when first introduced in 1964, there now exists a multitude of ethical guidelines that often contain conflicting standards (Emanuel 2013). In comparison to other guidelines and despite its expansion, the DoH remains a short document and lacks explanatory notes. Researchers and implementers of the DoH thus often lack access to the deliberative process that has shaped its principles, necessitating a focus on the ‘text which emerges rather than the debate which leads to the text’ when interpreting the DoH (Carlson et al. 2004, p. 696). Our analysis adopts this perspective, meaning that our primary interest in what follows is primarily conceptual rather than historical. Starting from the text itself, we examine how the concept of vulnerability has evolved through different revisions of the DoH, identifying shifts from implicit to explicit references and changes in how vulnerability is framed within the document. Additionally, we explore how these shifts reflect broader ethical discourses, particularly concerning vulnerability and the challenges of meeting the needs of vulnerable populations and individuals in medical research.

## Vulnerability in the Declaration of Helsinki

### The 1964 DoH (1964): vulnerability as an implicit concern

The DoH does not explicitly reference ‘vulnerability’ until its 5th revision in 2000, where the term is used to denote certain populations that require specific ethical considerations in research. Nevertheless, all early versions of the DoH arguably identify distinct classes of ‘non-paradigmatic’ (or ‘particularly vulnerable’) subjects that require specific safeguards or attention in research. In the 1964 DoH, there are three such groups: those susceptible to personality alteration due to drugs or experimental procedures (paragraph I.5), those incapable of giving or refusing consent (paragraphs II.1, III.3a, III.3b), and those in dependent relationships with investigators (paragraph III.4a). The first group is addressed in the basic principles section; the second both in the section on research combined with care and the section on non-therapeutic research; the third in the section on non-therapeutic research.

The 1964 DoH’s concern for personality alteration due to drugs or experimental procedures is, to our knowledge, a relatively unique issue in the literature on vulnerability. The issues surrounding consent and dependency, in contrast, emphasise the importance of a subject’s ability to exercise their power of choice. Paragraph III.3b states that the subject ‘should be in such a mental, physical and legal state as to be able to exercise fully his power of choice.’ While this statement might suggest that non-therapeutic research cannot be conducted on individuals with legal incapacity, paragraph III.3a in the same section states that consent from a legal guardian is required in cases of legal incapacity. Thus, there seems to exist a certain ambiguity on whether non-therapeutic research can, in fact, be conducted on legally ‘non-paradigmatic’ subjects.^13^

Paragraph III.4a addresses research subjects in dependent relationships to the investigator. The paragraph states that an investigator ‘must respect the right of each individual to safeguard his personal integrity, especially if the subject is in a dependent relationship to the investigator.’ By blending a categorical claim – the personal integrity of all research subjects (in non-therapeutic research) must be respected – with a comparative one – the personal integrity of dependent research subjects, in particular, requires special attention – the paragraph effectively foreshadows the tension between universal and circumstantial vulnerability discussed earlier. What the paragraph seems to suggest is that respecting a research subject’s personal integrity is, if not qualitatively, at least quantitatively, a different task when dealing with a dependent subject compared to a non-dependent subject.

By highlighting the dependency between a research subject and an investigator, the 1964 DoH anticipates later discussions on vulnerability in research ethics. In an influential background paper for a 2001 National Bioethics Advisory Commission (NBAC) report, Kipnis (2001) identifies six different types of vulnerability-inducing situations in which a research subject is open to being harmed or exploited by researchers. In what Kipnis calls deferential vulnerability, the subject’s decision-making is influenced by external parties even though those exerting the influence lack formal authority over the subject. The category also includes situations where potential subjects fear that their refusal to participate could negatively affect their care – a concern notably raised in the 1st revision of the DoH.

### 1st, 2nd, 3nd, and 4th revisions (1975–2000): Tokyo revision and minor adjustments

The 1st revision of the DoH, adopted in Tokyo in 1975, nearly doubled the length of the DoH and can be considered the most significant revision until the 5th revision in 2000 (Carlson et al. 2004; WMA 1975). In this revision, all three ‘particularly vulnerable’ groups identified in the 1964 DoH undergo some changes in status and emphasis. In addition, the 1st revision also introduces some new vulnerability concerns. As noted above, a new paragraph (II.4) in the section on research combined with care reflects concerns about deferential vulnerability stemming from fears of retribution: ‘[t]he refusal of the patient to participate in a study must never interfere with the doctor-patient relationship.’

In the 1964 DoH, the concern for personality alteration was clearly framed as a comparative issue, a source of situational vulnerability requiring ‘special caution’ (WMA 1964). In the 1st revision, the concern for personality alteration is universal in scope: ‘[e]very precaution should be taken to respect the privacy of the subject and to minimise the impact of the study on the subject’s physical and mental integrity and on the personality of the subject’ (WMA 1975; paragraph I.6). The issue of dependency is separated from personal integrity and linked explicitly to informed consent, now in the section on basic principles (paragraph I.10): ‘[w]hen obtaining informed consent for the research project the doctor should be particularly cautious if the subject is in a dependent relationship to him or her or may consent under duress’. In such cases, the DoH stipulates that consent should be obtained by another doctor not involved in the investigation. Lastly, the paragraph addressing incapacity (I.11) is expanded and relocated to the basic principles section, and now includes mental incapacity and minors in addition to legal and physical incapacity.^14^

In summary, the 1st revision of the DoH removes one ‘particularly vulnerable’ group highlighted in the 1964 DoH – those susceptible to personality alteration by drugs or experimental procedures – while simultaneously introducing new concerns related to consenting under duress or under fear of retribution. The issue of dependency is addressed within the basic principles section, where it is separated from concerns about integrity and explicitly linked to informed consent. Similarly, the paragraph addressing incapacity is expanded and relocated to the basic principles section.

As noted, the 1st revision of 1975 was the most substantial of the early revisions and nearly doubled the length of the DoH. The 2nd, 3rd, and 4th revisions (WMA 1983; WMA 1989; WMA 1996), in contrast, were relatively minor, with the majority of the implicit statements regarding vulnerability remaining largely unchanged. The only notable adjustment leading up to the 5th revision concerns minors. The 2nd revision introduces an addition to paragraph I.11 regarding the consent of minors: ‘[w]henever the minor child is in fact able to give a consent, the minor’s consent must be obtained in addition to the consent of the minor’s legal guardian.’ In the 5th revision, the wording of this statement is changed from consent to assent: ‘[w]hen a subject deemed legally incompetent, such as a minor child, is able to give assent to decisions about participation in research, the investigator must obtain that assent in addition to the consent of the legally authorized representative.’

### 5th revision (2000): vulnerable populations, special protection, and exploitation concerns

The 5th revision of the DoH, adopted in Edinburgh in 2000, is the first version of the DoH in which the term ‘vulnerability’ is used (WMA 2000). Five groups are explicitly mentioned. Paragraph 8 reads:

Medical research is subject to ethical standards that promote respect for all human beings and protect their health and rights. Some research populations are vulnerable and need special protection. The particular needs of the economically and medically disadvantaged must be recognized. Special attention is also required for those who cannot give or refuse consent for themselves, for those who may be subject to giving consent under duress, for those who will not benefit personally from the research and for those for whom the research is combined with care.

The paragraph is somewhat ambiguously written, creating uncertainty regarding the status of the mentioned groups. Vulnerability itself is not explained beyond the response it requires, namely ‘special protection’. However, paragraph 8 simultaneously introduces two arguably separate responses: ‘the recognition of particular needs’ and ‘special attention’. Does the recognition of particular needs and special attention form, or constitute a part of, special protection? Are the five groups mentioned consequently to be considered vulnerable? In a qualitative study of various ethics documents, Zagorac (2016) holds the word ‘disadvantaged’ to denote vulnerability in the 5th revision of the DoH, but not in the 6th revision. Similarly, Forster et al. (2001) interpret paragraph 8 in the 5th revision as implying that the five explicitly mentioned groups are indeed to be considered vulnerable. This interpretation is supported by the WMA’s suggested revisions for a preliminary draft of the 6th revision, publicly available on the website of the Nuffield Council on Bioethics (NCOB). In the draft in question, responded to by the NCOB in February 2007, the revised and arguably clearer paragraph 8 reads (NCOB 2007, emphasis added):

[…] Some research populations are vulnerable and need special protection. *These include* the educationally, economically or medically disadvantaged, those who cannot give or refuse consent for themselves, those who may be subject to giving consent under duress, and those for whom the research is combined with medical care.^15^

Nearly all of the groups mentioned in paragraph 8 are also referenced elsewhere in the document. Paragraph 23 reiterates concerns for those who may give consent under duress and, similar to earlier versions, raises concerns about those who are in a dependent relationship with the investigator. According to paragraph 23, consent should in such cases be obtained by another well-informed physician who is not engaged in the investigation – a measure arguably more appropriate for the latter group than the former. Paragraphs 24 and 26, in turn, delineate the conditions for conducting research on those who cannot consent for themselves. According to the 5th revision of the DoH, these groups should not be included in research unless the research is necessary to promote the health of the population represented and cannot be performed on legally competent persons. Lastly, the ethical conditions for conducting research on those for whom the research is combined with care is outlined in section C, ‘Additional Principles for Medical Research Combined with Medical Care’.

As noted, the focus on vulnerable groups (as opposed to vulnerable individuals) has been historically predominant in research ethics, and can be traced at least to the Belmont Report, which was published in 1978 (Luna 2009; National Commission for the Protection of Human Subjects of Biomedical and Behavioral Research 1978). In the Belmont Report, racial minorities, the economically disadvantaged, the very sick and those institutionalised are considered vulnerable (National Commission for the Protection of Human Subjects of Biomedical and Behavioral Research 1978). According to Nickel (2006), the Report uses vulnerability in two different senses which correspond to two fundamental ethical principles: respect for persons and justice. Respect for persons relates to the ability of a subject to provide autonomous informed consent, while justice relates to the fair distribution of the burdens and benefits in research. Using Nickel’s approach, one might argue that the 5th revision of the DoH applies a mainly ‘consent-based’ understanding of vulnerability, meaning that it understands vulnerability primarily through the principle of respect for persons, as a diminished ability to give voluntary and autonomous consent (Hurst 2008).^16^ Alternatively, one might contend that the list of vulnerable groups in the 5th revision lacks an organising principle (Zagorac 2016).

Like other contemporary guidelines, the 5th revision’s subpopulation approach to vulnerability was criticised due to its apparent broadness. In their article on the 5th revision, Forster et al. (2001, p. 1451) argue that the approach taken to vulnerability in the 5th revision essentially renders vulnerability meaningless:

[T]he new declaration goes further, making every conceivable person vulnerable, from patients with an illness, to those who cannot give consent, to healthy volunteers (provision 8; panel 2). The new declaration expands the category of vulnerability so broadly that it eliminates this category as a special protection; if everyone is vulnerable, no one is entitled to special protection.

Similar critiques of the subpopulation approach to vulnerability have since become common in the literature.^17^ In perhaps the most influential of these, Levine et al. (2004, p. 46) argue that if nearly everyone is vulnerable, vulnerability is a concept ‘too nebulous to be meaningful’. First, insofar as virtually any person belongs to a group that may reasonably be considered vulnerable, the concept is ineffective for providing specific protections. Second, the nearly sole focus on group traits that supposedly compromise the ability to provide consent diverts attention from aspects inherent to the research itself or the broader institutional, social, and economic circumstances that place the subject at a higher risk for harm or wrong. Third, the concept of vulnerability functions in a stereotyping manner, labelling entire groups without acknowledging individual differences between individuals in said groups. Perhaps reflecting this criticism, research guidelines and ethical documents have generally shortened their lists of vulnerable groups, or stopped identifying groups altogether. As we will see, the 6th revision of the DoH identifies only two vulnerable groups, while the 7th and 8th revisions identify none.

At the time of publication, the most controversial paragraphs were paragraphs 29 and 30 in section C. Paragraph 29 stipulates that a new intervention should be tested against the best current prophylactic, diagnostic, and therapeutic methods, thus seemingly ruling out the use of placebos when a proven treatment for the condition in question exists (Carlson et al. 2004).^18^ Paragraph 30, in turn, states that after the trial, every participating subject should be assured access to the ‘best proven’ prophylactic, diagnostic, and therapeutic methods identified by the study.

The position of the 5th revision on the use of placebos does not significantly differ from the overall guidance on the use of placebos provided in the 4th revision (ibid.). Nevertheless, paragraph 29 was met with harsh criticism, especially from the United States, where it was framed as an ‘attack on the use of placebos’ that would complicate drug efficacy evaluation and drive up the costs of drug development (Wolinsky 2006, p. 670). The US Food and Drug Administration (FDA), which had cited the DoH since 1975, started referring to the 3rd revision from 1989 as a way of sidestepping the new 5th revision (ibid.) The increased criticism led the WMA to issue a note of clarification to paragraph 29 in 2001 – the first time the WMA released explanatory text to shed light on the interpretation of a specific paragraph (Carlson et al. 2004). The note of clarification states that the use of placebos may be acceptable when: a) there is a ‘scientifically compelling’ reason, or b) the condition studied is minor, and the patients receiving placebo will not be subject to additional risk of serious harm (WMA 2004).

The question in paragraph 29 is whether participants are worse off in a trial than before a trial, while the question in paragraph 30 is whether participants are worse off after the trial than they were during it (Carlson et al. 2004). Thus, both paragraphs are tightly linked to vulnerability. Questions of the burdens and benefits of research were of growing importance at the time of publication of the 5th revision of the DoH due to an increased amount of research conducted in resource-poor countries (Emanuel et al. 2004; Macklin 2001; Vastag 2000). In contrast to the contemporary CIOMS guidelines of 2002, however, the 5th revision of the DoH did not identify certain populations in low and middle-income countries (LMICs) as vulnerable. This position was criticised for not going far enough in addressing ‘the profoundly altered landscape’ of research involving human subjects (Eckenwiler et al. 2008, p. 765).

### 6th revision (2008): ‘particularly’ vulnerable populations, coercion, and undue influence

The 6th revision of the DoH, published in Seoul in 2008, introduces significant changes to both the status and formulation of vulnerability (WMA 2008). Notably, the period between the 5th and 6th revisions saw UNESCO member states adopt the Universal Declaration on Bioethics and Human Rights, described as ‘the most notable acknowledgment of human vulnerability’ (Haugen 2010, p. 210; UNESCO 2005). The UNESCO Declaration seeks to reconcile both universal and circumstantial vulnerability, and considers vulnerability the source of a variety of diverse concerns and implications (IBC 2013; Bracken-Roche et al. 2017). Similarly, the 6th revision of the DoH marks the first time vulnerability is mentioned in the section on general principles, thereby elevating it from an application of other fundamental principles to a fundamental principle in its own right (Bracken-Roche et al. 2017; ten Have 2016; WMA 2008). Additionally, the introductory paragraph on vulnerability (paragraph 9) now speaks of ‘particularly vulnerable’ populations when discussing those in need of special protection, a terminological shift that suggests a broader awareness of universal vulnerability. The 6th revision thus aligns with the growing tendency to view vulnerability as a markedly broader concept while still emphasising the need for special protection for certain groups. However, since the prefix ‘particularly’ is later omitted in paragraph 17, where vulnerability is juxtaposed with the undefined term ‘disadvantaged’ populations, the 6th revision also inadvertently introduces some conceptual ambiguity.

Paragraph 9 specifies by way of example two criteria for particular vulnerability: the inability or refusal to give consent and susceptibility to coercion and undue influence. These criteria correspond to two of the groups identified in the 5th revision, namely those who cannot give or refuse consent for themselves and those who may be subject to giving consent under duress (Zagorac 2016). The introduction of ‘coercion’ and ‘undue influence’ as specifications of what vulnerable populations should be protected against initiates a shift from ‘protection of’ to ‘protection against’ that culminates in the 7th and 8th revisions defining vulnerability more broadly as a higher risk for wrongs (ibid.). In its entirety, paragraph 9 of the 6th revision reads:

Medical research is subject to ethical standards that promote respect for all human subjects and protect their health and rights. Some research populations are particularly vulnerable and need special protection. These include those who cannot give or refuse consent for themselves and those who may be vulnerable to coercion or undue influence.

Paragraph 17, in turn, reads:

Medical research involving a disadvantaged or vulnerable population or community is only justified if the research is responsive to the health needs and priorities of this population or community and if there is a reasonable likelihood that this population or community stands to benefit from the results of the research.

Although the list of explicitly mentioned vulnerable groups is reduced to two, a closer analysis shows that the 6th revision of the DoH implicitly covers the vulnerability of all the groups included in the 5th revision, except for those who are economically disadvantaged. The new section on additional principles for medical research combined with care aligns with the call for the special protection of those for whom research is combined with care in the 5th revision. The vulnerability of those who will not personally benefit from the research is addressed in articles 14 and 33, which refer to post-trial access to interventions identified as beneficial in the study, and in paragraph 17, which stipulates that the health needs and priorities of the population or community must be considered and that there must be a reasonable likelihood that they will benefit from the research. Arguably, these provisions also touch upon the vulnerability of the medically disadvantaged – a group that may also partly align with the 6th revision’s concern about underrepresented populations in medical research (paragraph 5; see below). Therefore, one could argue that the concern for vulnerability remains quite consistent between these two revisions of the DoH – except for those economically disadvantaged.

Paragraph 5, which states that medical research ultimately must involve research on human subjects, now additionally states that populations that have historically been understudied should be provided appropriate access to participation. This is a concern highlighted both in the literature on vulnerability and the comments on the 5th revision of the DoH. Eckenwiler et al. (2008, p. 765) criticise the 5th revision of the DoH for not linking vulnerability explicitly more tightly with justice and retaining a view of vulnerability that is ‘merely protectionist’. From the lens of justice, questions about underrepresentation and the sharing of benefits are as important as the burdens of research, and exclusion in the name of protection may be a particular source of vulnerability (ibid.; Rogers et al. 2012). As noted, such concerns have been raised regarding children, pregnant women, and the elderly (Blehar et al. 2013; Chambers et al. 2008; Langmann 2023; Lyerly et al. 2008; Macklin 2010; Schonfeld 2013; Schwenzer 2008; van der Zande et al. 2017).

Overall, the changes made in the 6th revision may be described as somewhat ambivalent. On the one hand, the statements on vulnerability in paragraph 17 of the principles section link vulnerability more closely with justice, elevating it to the level of a fundamental principle. Paragraph 5 now addresses populations historically underrepresented in research, and the use of the term ‘particularly vulnerable’ populations in paragraph 9 reflects the growing tendency to view vulnerability as a markedly broader concept. On the other hand, the two examples of vulnerable groups now link vulnerability to concerns exclusively related to a diminished capacity to give voluntary consent, namely undue influence and coercion. The introduction of these terms raises additional interpretive challenges, as does the rather confusing mention of ‘disadvantaged populations’ alongside ‘vulnerable populations’ in paragraph 17.

### 7th revision (2013): vulnerable individuals and wrongs

The 7th revision of the DoH was adopted in Fortaleza, Brazil, in October 2013 (WMA 2013). A key change in this revision is the explicit linking of particular vulnerability to an increased likelihood of being wronged or harmed. Additionally, the revision references vulnerable individuals for the first time. Paragraph 19 reads:

Some groups and individuals are particularly vulnerable and may have an increased likelihood of being wronged or of incurring additional harm. All vulnerable groups and individuals should receive specifically considered protection.

The formulation of vulnerability in paragraph 19 partly aligns with an influential account presented by Hurst (2008) and further elaborated by Martin et al. (2014). Hurst (2008) reviews various definitions of vulnerability and finds them either too broad or too narrow. Consent-based definitions focus on the capacity to protect one’s interests but are limited to issues of consent. Harm-based definitions emphasise susceptibility to additional harms but may overlook non-physical wrongs. Comprehensive definitions attempt to cover both consent and fairness but may still miss aspects like the risk of disclosing confidential information. Ultimately, Hurst argues for defining vulnerability as an increased likelihood of incurring additional or greater wrong, including wrongful harm. The definition incorporates the idea that ‘even participants who benefit, and who suffer little or no physical harm, may be wronged if they lack information essential to their decisions’ (Henderson et al. 2007, p. 1736). Such wrongs are typically referred to as dignitary injuries (ibid.).^19^

The broader conceptualisation of vulnerability in terms of wrongs allows for a more comprehensive understanding of the various ways research subjects can be vulnerable (Bracken-Roche et al. 2017). As the DoH does not specify the specific nature of these wrongs, the shift furthermore allows for more nuance in the way the protection itself is understood. Insofar as vulnerability is not explicitly linked to decisional capacity, the implications of vulnerability may focus on the need for responsive research, justifications for both exclusion and inclusion, and the distribution of the risks and benefits of research at both individual and group levels. The revised paragraph 20 (paragraph 17 in the 6th revision, see p. 21) elaborates on some of these implications of vulnerability:

Medical research with a vulnerable group is only justified if the research is responsive to the health needs or priorities of this group and the research cannot be carried out in a non-vulnerable group. In addition, this group should stand to benefit from the knowledge, practices or interventions that result from the research.

Similarly to the approach taken for research involving persons incapable of giving consent in earlier versions, the revised paragraph 20 now requires that research on vulnerable populations is justified only if it cannot instead be conducted on a non-vulnerable group. Additionally, the ways in which vulnerable populations might benefit from the research results have been further elaborated. The 6th revision stated that vulnerable populations or communities should have a ‘reasonable likelihood’ of benefiting from the research results. The 7th revision broadens this perspective by referring to ‘knowledge, practices, or interventions that result from the research’ while simultaneously tightening the ‘reasonable likelihood’ criterion to state that these populations ‘should stand to benefit’.

Notably, the revised paragraph removes the confusing reference to ‘disadvantaged populations’ and the additional mention of ‘vulnerable communities’, which were juxtaposed with ‘vulnerable populations’ in the 6th revision. Whereas the 6th revision referenced vulnerable populations, disadvantaged populations, and vulnerable communities somewhat haphazardly, the 7th revision consistently uses the terms ‘vulnerable groups’ or ‘vulnerable individuals’. While the terminological differences between population, group, and community may seem minor, the 7th revision of the DoH thus arguably reads as a more coherent document regarding vulnerability overall. Nevertheless, the prefix ‘particularly’ in paragraph 19, similarly to the 6th revision, still creates some unnecessary conceptual ambiguity as it is neither explained nor used elsewhere in the document.

Some other changes to the DoH are of major relevance from a vulnerability standpoint. First, paragraph 34 broadens the responsibility of research sponsors, researchers, and host governments to make post-trial access available to participants in the trial. Second, paragraph 15 stipulates a wholly new responsibility for compensation and treatment of subjects who have been harmed as a result of participation in a trial. The 6th revision required that information regarding compensation be stated in the research protocol but did not require compensation. Lastly, paragraph 15 acknowledges the importance of bodily integrity and financial interests beyond autonomy being respected.

As a whole, the changes introduced in the 7th revision represent an attempt to provide a more comprehensive and justice-oriented approach to both vulnerability and medical research as a whole, acknowledging the importance of distributive justice and beneficence in addition to questions relating to patient autonomy (Malik and Foster 2016).

### 8th revision (2024): vulnerable situations and the risks of exclusion

The WMA announced the adoption of the latest, 8th revision of the DoH in Helsinki, Finland, in October 2024 (WMA 2024a). The accompanying press release states that the revision provides ‘increased protection for vulnerable populations […] and stronger commitments to fairness and equity in research’ (WMA 2024b). According to the chair of the revision workgroup, Dr. Jack Resneck Jr., the 8th revision attempts to ‘address the theme of distributive and global justice [and] calls on researchers to carefully consider how the benefits, risks, [and] burdens of research are distributed’ (ibid). An updated paragraph 6 reflects this systemic focus, stating:

Since medical research takes place in the context of various structural inequities, researchers should carefully consider how the benefits, risks, and burdens are distributed.

This addition reflects the 8th revision’s broader ambition to incorporate systemic inequities into ethical considerations. One of the most prominent changes to the document is the replacement of the term ‘subjects’ with ‘participants’ throughout, signaling a shift toward recognising participants as active partners in research (ibid.). Similarly, gendered language has been removed to promote inclusion.

Vulnerability is again treated under its own subheading in paragraphs 19 and 20, now titled ‘Individual, Group, and Community Vulnerability.’ The extended paragraph 19 reads:

Some individuals, groups, and communities are in a situation of more vulnerability as research participants due to factors that may be fixed or contextual and dynamic, and thus are at greater risk of being wronged or incurring harm. When such individuals, groups, and communities have distinctive health needs, their exclusion from medical research can potentially perpetuate or exacerbate their disparities. Therefore, the harms of exclusion must be considered and weighed against the harms of inclusion. In order to be fairly and responsibly included in research, they should receive specifically considered support and protections.

The revised paragraph 20 in turn reads:

Medical research with individuals, groups, or communities in situations of particular vulnerability is only justified if it is responsive to their health needs and priorities and the individual, group, or community stands to benefit from the resulting knowledge, practices, or interventions. Researchers should only include those in situations of particular vulnerability when the research cannot be carried out in a less vulnerable group or community, or when excluding them would perpetuate or exacerbate their disparities.

The revised paragraph 20 reintroduces the notion of vulnerable communities from the 6th revision, a move which according to Reis et al. (2025) represents ‘a more communal approach to research, highlighting the principles of solidarity and reciprocity’ (Reis et al. 2025, p. 21). The second part of the paragraph also introduces a new criterion for conducting research involving those in situations of particular vulnerability – namely when excluding them would perpetuate or exacerbate disparities. We may note that the terminology in paragraphs 19 and 20 differs regarding particular vulnerability; paragraph 19 refers to ‘more’ vulnerability, while paragraph 20 uses the term ‘particular’ vulnerability. This inconsistency in terminology arguably continues to weaken the internal coherence of the document, albeit less starkly than in the 6th and 7th revisions.

More significantly, the phrasing in paragraph 19 explicitly links vulnerability to a research setting and the role of being a research participant. The formulation in paragraph 19 – ‘situation of more vulnerability as research participants’ – treats vulnerability as an essentially relational property, that is, a relationship between the individual, group or community and the research setting. On a general level, the formulation aligns with Florencia Luna’s (2019) metaphor of vulnerability as being constituted by layers that are activated by specific external conditions, or ‘stimulus conditions.’ For example, invasive procedures might activate layers related to physical risk, while trials conducted in resource-poor settings may activate layers tied to systemic inequities. The 8th revision of the DoH organises its ‘layers’ into two main groups: fixed layers, and context-dependent and dynamic layers. This distinction echoes other attempts to distinguish between sources of vulnerability that are inherent or fixed and sources that are extrinsic or situational (Rogers & Ballantyne 2008; see also Dunn et al. 2008). The phrasing in paragraph 19 suggests these categories are intended to be mutually exclusive. However, such a distinction is not easily made, as any individual characteristic necessarily expresses itself in the context of social circumstances (see for example Herring 2016, p. 27–28; Luna 2015).

The context-dependent framing of vulnerability in the 8th revision naturally avoids the pitfalls of categorical labelling, as the locus of vulnerability shifts from characteristics of the research subject to the research context and its corresponding harms or wrongs. However, it simultaneously creates a subtle tension with the exclusion-related concerns raised in paragraphs 19 and 20, which address broader systemic issues that generally transcend the immediate research context. Resneck (2025, p.16), describes the rationale behind the exclusion-related concerns followingly:

At several meetings focused entirely on this topic, impassioned and consistent feedback contended that starting with a default of exclusion for all those in positions of vulnerability had resulted in enormous gaps in medical knowledge about certain populations (especially women, children, and racial and ethnic minoritized groups) and exacerbated disparities.

A further example concerns the elderly, a group often considered particularly vulnerable in public and academic debates (Bozzaro et al. 2018). While the elderly are not necessarily particularly vulnerable per se simply because of their old age – meaning that not every elderly person should automatically be considered particularly vulnerable – they also cannot be reduced to a scattered group of unrelated individuals (Macioce 2023). There is a common theme to old age that serves as a salient warning sign for special attention and for considering whether a particular elderly individual may be more susceptible to harm or wrongs (Bozzaro et al. 2018). This theme encompasses certain social realities, such as decisions around resuscitation, prescribing practices, and conditions in nursing homes – patterns that arguably reflect broader societal inequities and often extend beyond both research settings and the healthcare context. It involves not only the physical and cognitive dimensions associated with aging but also the economic and social dimensions that position the elderly similarly within society and expose them to ‘analogous conditions of vulnerability’ across various contexts (Macioce 2023, p. 219). Addressing the vulnerabilities of the elderly thus requires recognising the shared layers of old age that may amplify susceptibility to harm while avoiding essentialist assumptions or reducing elderly individuals to passive victims (ibid.).

Another way of framing this tension is by asking whose harm or wrongs is being addressed in paragraph 19 and 20. The traditional harms and wrongs associated with research inclusion involve risks tied to the specific research context, such as exploitation or a higher risk of adverse effects and are primarily experienced by the individual participating. By contrast, the effects of exclusion (e.g. disparities) operate primarily at a group level and often extend beyond the immediate research environment, both temporally and geographically. These effects are not primarily experienced by the individual excluded – at least insofar as research participation genuinely entails uncertainty about the effectiveness of treatment – but rather by those affected by the lack of scientific knowledge that the exclusion results in.

The challenges of balancing broader, group-level concerns with the individual risks of participating in research are naturally not unique to the DoH. Nor are they challenges that will be resolved through conceptual analysis of vulnerability alone. However, it is worth noting that recent conceptual work on vulnerability has illuminated how inherent dispositions, interpersonal relationships, and larger structural processes both produce, alleviate, and exacerbate our vulnerabilities, thereby increasing our susceptibility to certain harms or wrongs (Mackenzie et al. 2014; ten Have 2016). Turner (2006) introduces the concept of ‘institutional precariousness,’ arguing that dysfunctional institutions can serve as sources of vulnerability. Rogers et al. (2012) speak of ‘pathogenic vulnerabilities’, referring to vulnerabilities that are produced or worsened by interventions or policies designed to address vulnerability – blanket exclusion from clinical research, for example. Pathogenic vulnerabilities are especially troubling because they are not confined to a single research context but are rooted in broader structural injustices, such as oppression, marginalization, or systemic neglect. An example from clinical research concerns pregnant women, who, due to gaps in scientific knowledge, may face increased exposure to risk in both research participation and clinical care (Van der Zande et al. 2017). However, the concept of pathogenic vulnerability, as understood in the literature, does not align neatly with the DoH’s framing of vulnerability as context-dependent and specific to research participation. By anchoring vulnerability explicitly to the research setting – ‘as research participants’ – the DoH arguably limits the scope of its vulnerability framework to situational harms and wrongs within specific research contexts. Notably, the exclusion-related concerns in paragraph 19 are framed in terms of ‘disparities,’ a term that is neither restricted to health nor explicitly defined in the document.

To summarise, the 8th revision of the DoH introduces significant changes to the framing of vulnerability by emphasising its context-dependent, research-specific nature. This framing shifts the locus of vulnerability even further from inherent traits to the interaction between participants and the research context. However, the exclusion-related concerns raised in paragraphs 19 and 20 simultaneously create a subtle tension between this narrowed focus and broader systemic concerns, such as the underrepresentation of certain groups in research. By conflating group-level vulnerabilities and systemic injustices with individual-level risks of harm, the DoH arguably risks obscuring the ethical distinctions needed to effectively address these challenges.

## Discussion

Our analysis points us toward a DoH increasingly concerned with human vulnerability. From the initial implicit acknowledgments in the early versions to the more detailed and explicit mentions in subsequent revisions, the DoH has progressively emphasised the need for nuanced protection of research subjects. Over the six decades of the DoH’s existence, only one ‘vulnerability concern’ seems to have been completely dropped: the original 1964 DoH’s concern over those whose personality is liable to be altered by drugs or experimental procedures. Simultaneously, new concerns have been introduced with almost every single revision.

Vulnerability has nevertheless proven difficult to conceptualise coherently within the DoH itself. The document’s explicit statements on vulnerability have often struggled to keep pace with the growing recognition of the multifaceted and context-dependent nature of vulnerability in research. Policy documents now increasingly recognise that managing vulnerability requires an understanding of the specific dangers or threats involved (Zagorac 2016). Early versions of the DoH did not explicitly mention vulnerability but clearly identified certain populations requiring special ethical considerations in research. Initially, this ‘vulnerability concern’ was focused primarily on capability, meaning that individuals deemed unable to protect their own interests – particularly in terms of informed consent – required special protection or attention. This framework was carried over to the 5th revision in 2000, which, for the first time, explicitly listed vulnerable groups: (1) the economically and medically disadvantaged; (2) those who cannot give or refuse consent for themselves; (3) those who may be subject to giving consent under duress; (4) those who will not benefit personally from the research; and (5) those for whom the research is combined with care. While this list can still be read primarily as a conventional ‘consent-based’ categorisation of vulnerable subjects (see note 16), the inclusion of group (4) renders it somewhat haphazard and lacking an organising principle (Zagorac 2016). Consequently, the 6th revision included only two exclusively ‘consent-based’ groups. Notably, the scope of protection nevertheless remained roughly consistent: the only group omitted in the 6th revision was the economically disadvantaged, with specific protections for all other groups detailed elsewhere in the text (see p. 21–22).

From this perspective, the 6th revision’s focus on undue influence or coercion as the specific threats that vulnerable subjects should be protected from may be seen as an attempt to streamline the conceptualisation of vulnerability while still maintaining the general level of detailed protection. The 6th revision initiated a shift from ‘protection of’ (the vulnerable) to ‘protection against’ (some harm or wrong), a change generally considered to result in more context-dependent and better-tailored protection, thereby reducing blanket exclusion (ibid.). The conceptual clarity nevertheless came at a cost: the framing of vulnerability as an application of the principle of respect for autonomy arguably rendered vulnerability a somewhat immaterial phenomenon. Insofar as wrongs such as coercion and undue influence are already protected against by a functioning consent process, the designation of someone as vulnerable was reduced to something akin to a warning sign rather than a fundamental principle in its own right.

The 7th revision’s formulation of vulnerability as linked to the overarching concept of wrongs provided a much-needed anchoring to its ethical application. The broadness of the formulation enables a more comprehensive understanding of the various ways in which research subjects can be vulnerable without predefining the necessary responses to prevent additional wrong or harm (Bracken-Roche et al. 2017). The lack of specificity regarding the wrongs themselves further gives the DoH an aura of ‘tentative immortality’, which Emanuel (2013, p. 1533) advocated the DoH should strive for. However, whether a wrongs-based formulation is compatible with vulnerability as a fundamental, non-reducible ethical principle remains unclear. Critics such as Anthony Wrigley (2015, p. 486) note that the underlying ethical concern in such formulations seem to be grounded in ‘lists of identifiable wrongs’, which seems to presuppose a moral framework of wrongs and wronging not linked to vulnerability itself. In contrast, the philosophical literature on vulnerability generally holds our vulnerability to the actions of others to be the primary source of moral obligation (Goodin 1985; Mackenzie 2016). Thomasma (2000, p.8), for example, formulates a principle of vulnerability which states: ‘[i]n human relations generally, if there are inequities of power, knowledge, or material means, the obligation is upon the stronger to respect and protect the vulnerability of the other, and not to exploit the less advantaged.’ In this view, vulnerability itself is a ‘morally salient characteristic of humanity that ought to play a role in guiding our actions and deliberations’ (Mackenzie 2016, p. 91).^20^

The 7th revision also moved away from viewing vulnerability primarily as a problem of individual decision-making and instead connected it more closely with justice, thereby lessening the ‘merely protectionist’ approach for which the 5th revision was criticised (Eckenwiler et al. 2008, p. 765). Historically, research ethics has emphasised immediate protections in specific contexts and the avoidance of additional harm. While effective for addressing short-term risks, this situational focus risks neglecting the root causes – such as economic disparities or social inequities – that create, perpetuate or exacerbate vulnerability. The 8th revision’s explicit inclusion of structural inequities in paragraph 6 and the revised paragraphs on vulnerability represent important steps toward addressing such concerns. By calling on researchers and ethics committees to actively consider how their decisions contribute to or mitigate systemic inequities (Resneck 2025), the DoH acknowledges that research ethics must extend beyond the protection of participants within discrete contexts, making vulnerability a central concept for future revisions of the DoH. However, this broadened perspective also presents significant challenges that future revisions must meet to ensure that the document remains internally coherent and practically applicable for researchers and research ethics committees alike.

### Towards the 9th revision

Vulnerability has become a central notion in the DoH and is likely to take on even greater significance in future revisions. Given the more than decade-long interval between the 7th and 8th revisions – more than double the five years that separated the 6th and 7th – it is reasonable to assume that the medical landscape will have evolved substantially by the time the 9th revision is published. Rapid advancements in technology are one area likely to shape future discussions. For instance, the digitalisation of health services risks exacerbating digital inequality (Kaihlanen et al. 2022), while emerging technologies such as health-related artificial intelligence (AI) may introduce novel ethical challenges. Shaw (2025) highlights significant variability in AI literacy among researchers, ethics committees, and the public, as well as the lack of clarity regarding present and future harms associated with AI in health research. These harms may disproportionately affect communities already facing structural disadvantages in relation to these technologies (ibid.).

Notwithstanding the new ways in which technology may exacerbate vulnerability and disparities, the primary challenge for future revisions regarding vulnerability lies in integrating an increasingly context-dependent framework of vulnerability with the theme of distributive justice, both conceptually and practically. The 8th revision frames vulnerability primarily as contingent on situational factors within specific research contexts rather than as a fixed characteristic. However, systemic inequities – such as those driving the underrepresentation of certain populations in research – often transcend these contexts. Addressing such vulnerabilities requires recognising the shared layers or analogous conditions that perpetuate them, and a framework to articulate them. Categorical labelling and essentialist assumptions risk undermining their intended purpose. As the DoH’s concern with exclusion demonstrates, actions designed to protect vulnerable subjects can, paradoxically, perpetuate vulnerability or generate new ones.

While the use of extensive lists of vulnerable subjects has been widely criticised, making it unlikely that the DoH will reinstate such lists, it is worth noting that the concept of (group) vulnerability was originally introduced to address systemic injustices (ten Have 2016). Recent research suggests that a group-based approach to vulnerability need not be inherently reductionistic or essentialist (Macioce 2023). In other words, a group-level approach to vulnerability does not necessarily imply rigidity or stereotyping (Bozzaro et al. 2018). For instance, the CIOMS guidelines of 2002 retain a conceptual distinction between vulnerable groups, the specific individuals within them, the reason for their vulnerability, and the appropriate response.^21^

In recent years, a growing body of literature has refined how vulnerability is conceptualised and applied in research ethics (Armstrong 2017; Boldt 2019; Bracken-Roche et al. 2017; Hurst 2008; Luna 2009; Luna 2019; Martin et al. 2014; Rogers et al. 2012; ten Have 2015; ten Have 2016). One important lesson is that focusing exclusively on individual transactions – for example, between researcher and subject – may obscure broader systemic processes that create and sustain vulnerability, especially in a global context (ten Have 2016). To the extent that vulnerability is created, perpetuated, or exacerbated at a systemic level, it cannot be fully addressed by merely empowering individuals or safeguarding their autonomy-enhancing options (ten Have 2015). Consequently, vulnerability challenges traditional bioethical principles, making it a ‘troubling’ yet vital issue in global bioethics (ibid., p. 403).

### Study limitations

This work is a conceptual analysis. It relies on the synthesis and interpretation of existing literature and the various revisions of the DoH. Its insights are necessarily contingent on the scope and quality of the reviewed literature, as well as the interpretive framework applied.

## Conclusion

Vulnerability has become a central notion in the DoH and is likely to take on even greater significance in future revisions. From the initial implicit acknowledgments in the early versions to the more detailed and explicit mentions in subsequent revisions, the DoH increasingly emphasises the need for detailed protection of research subjects. Additionally, the DoH has progressively prioritised distributive justice and the need to address systemic inequities as central ethical concerns in medical research. This evolution culminates in the 8th revision, which explicitly acknowledges that research occurs within the context of various structural inequities and underscores the need for the responsible inclusion of vulnerable subjects to reduce disparities. By broadening its scope to encompass these systemic concerns, the 8th revision emphasises the societal impact of research practices and situates vulnerability within a broader framework of justice.

Vulnerability has nevertheless proven difficult to conceptualise coherently within the DoH itself. The document’s explicit statements on vulnerability have often struggled to keep pace with the growing recognition of the multifaceted and context-dependent nature of vulnerability in research. The 8th revision frames vulnerability primarily as contingent on situational factors within specific research contexts rather than as a fixed characteristic. However, systemic inequities – such as those driving the underrepresentation of certain populations in research – often transcend these contexts. Addressing such vulnerabilities requires recognising the shared layers or analogous conditions that perpetuate them and a framework to articulate them. Future revisions of the DoH face the challenge of integrating vulnerability in an internally coherent and practically applicable way for both researchers and ethics committees.

## Data Availability

No datasets were generated or analysed during the current study.

## Footnotes

1 The Merriam-Webster Dictionary defines vulnerability as being ‘capable of being physically or emotionally wounded’. Similarly, the Oxford Dictionary of English defines vulnerability as being ‘exposed to the possibility of being attacked or harmed, either physically or emotionally’.

2 The claim that everyone is vulnerable holds significance beyond research ethics (see for example Herring 2016; ten Have 2016). Insofar as every human being is vulnerable, the baseline frame of reference of a ‘paradigmatic research subject who has all the characteristics of a well-informed, rational, competent, self-determining agent’, is put in doubt (ten Have 2015, p. 400). Thus, the accuracy of traditional divisions in legal and social contexts is questioned. Ten Have (ibid.) further notes that in its most radical interpretation, as expressed by philosophers such as Levinas, vulnerability implies that our being in the world is always a ‘being-together’, where ‘the ethical relationship of responsibility between human beings is prior to self-interest and individual choice’. In this view, vulnerability and relatedness are not so much attributes of a subject as what constitutes it: ‘[i]t is not that we have selves that join together to form relationships, but it is out of our relationships that the self exists’ (Herring 2016, p. 12). Thus, the notion of universal vulnerability and its implications can be portrayed as a challenge to contemporary, liberal individualist bioethics (ten Have 2015).

3 The notion of universal vulnerability has occasionally been criticised for being so broad and vague as to be near meaningless (Luna 2009; Wendler 2017). However, as Herring (2016, p. 23) notes, ‘[o]ne can sensibly claim that we are all vulnerable, while at the same time accepting that some are in particularly vulnerable situations and therefore deserve especial attention’ (Herring 2016, p. 23).

4 In what follows, the term ‘subpopulation approach’ refers (broadly) to any approach to vulnerability that primarily aims at identifying and categorising specific groups of individuals as vulnerable based on some shared characteristic or circumstance. See Luna (2019).

5 Specifically, modern bioethics often conceptualises vulnerability as comprising three key characteristics: dynamicity, context-dependency, and individualisation. Dynamicity generally refers to how vulnerability evolves over time, reflecting changes in an individual’s or group’s circumstances. This can be thought of as the temporal flow of vulnerability, where factors such as health, autonomy, or social standing can shift throughout a study or a lifetime. Context-dependency, by contrast, generally refers to how vulnerability shifts across different settings or environments. This can be conceptualised as the situational landscape of vulnerability, emphasising that individuals or groups may be more or less vulnerable depending on external factors such as healthcare access, cultural barriers, or research design. For example, a person might be more vulnerable in a rural healthcare setting with limited resources compared to an urban one with greater support. Lastly, individualisation refers to the assumption that the locus of vulnerability is – or should be – the individual, rather than any particular group to which the individual belongs (or be interpersonal in nature). Thus constructed, individualisation represents the true locus or ‘frame’ of vulnerability.

6 See Martin et al. (2014) for a detailed account of the linkages between vulnerability, harm, and wrongs.

7 While the Nuremberg Code is often credited as the first guideline to establish ethical regulations based on the principle of informed consent, its scope and status in the history of research ethics remain subjects of debate (Moreno et al. 2017; Vollman and Winau 1996). In addition to being written as part of a trial judgement, it remained unknown in many countries, had limited impact on practice, and was not adopted by any government – with the partial exception of the US Department of Defense in 1953 (Moreno et al. 2017; Ehni and Wiesing 2024).

8 For an overview of the DoH’s influence on international and national legal instruments and other guidelines on research ethics during the first four decades of its existence, see Human and Fluss (2001). For an analysis of the DoH and the law, see Sprumont et al. (2007).

9 Quoted in Human and Fluss (2001). Writing in 2008, Ashcroft (2008) argues that the authority of the DoH has diminished since 2000. However, he simultaneously notes that it seems hard to conceive of a competing guideline with similar authority replacing the DoH.

10 The 8th revision thus addresses long-standing criticisms regarding the DoH’s audience. Millum et al. (2013) previously noted contradictions in the document’s stated audience, as certain paragraphs clearly targeted non-physicians, despite its focus on physicians. They argued that the validity of ethical principles rests on their universal applicability rather than the identity of their authors: ‘a statement of ethical principles does not require a mandate from the people who ought to follow those principles’ (ibid., p. 2143). The updated DoH explicitly extends its principles to all individuals, teams, and organisations involved in medical research, and it amends its language to better reflect the interdisciplinary reality of research. For further discussion, see Emanuel (2013), Ehni and Wiesing (2019), and Resneck (2025).

11 The idea of comparing the revisions of the DoH by word count and article count is borrowed from Emanuel (2013).

12 See also Eriksson et al. 2008.

13 The requirement for a subject to be in such a mental, physical and legal state as to be able to fully exercise his or her power of choice was omitted in the 1st revision of 1975.

14 The early versions of the DoH seem to view the vulnerability of minors primarily as a special form of legal incompetence. This is most obvious in the 2000 version of the DoH, in which minors are mentioned in two paragraphs, 24 and 25. Paragraph 24 reads: ‘For a research subject who is legally incompetent, physically or mentally incapable of giving consent or is a legally incompetent minor, the investigator must…’ Paragraph 25 reads: ‘When a subject deemed legally incompetent, such as a minor child…’.

15 The WMA’s explanatory comments on the suggested revision read: ‘Minor grammatical changes. The deletion near the end [concerning those who will not benefit personally from the research] incorporates the idea that, by its very nature, research cannot guarantee that participants will benefit from the intervention.’.

16 The 5th revision’s focus on respect for persons is particularly clear in the proposed draft for the 6th revision (see p.16), in which the only clearly ‘justice-based’ group, namely those who will not benefit personally from the research, is no longer included. All other populations mentioned in the 5th revision have historically been associated with challenges to the procedure of informed consent. According to Beauchamp and Childress’ (2019) widely recognised approach to informed consent, valid informed consent has competence and voluntariness as preconditions and additionally consists of disclosure, understanding, decision, and authorisation. In this reading, the DoH’s group of those who cannot give or refuse consent for themselves refers to subjects with either limited capacity or freedom to consent, for example, children, prisoners, and persons with mental or behavioural disorders, whose competence can be doubted (the CIOMS guidelines of 2002 refer to this group as the ‘conventional’ group of vulnerable subjects). Those medically disadvantaged refer to subjects with serious, potentially disabling or life-threatening diseases, whose voluntariness can be questioned (Nickel 2006). Similarly, the voluntariness aspect of informed consent could be questioned with regards to the economically disadvantaged, where the concern is mainly undue influence. Those who consent under duress refer to persons who may be coerced to consent. Finally, those for whom research is combined with care would be included because of the risk of the ‘therapeutic misconception’, whereby research subjects conflate the imperatives of clinical research with those of clinical care, potentially overestimating the benefits and underestimating the risks of participation. The therapeutic misconception is usually framed as a problem for either the understanding component or the appreciation component of informed consent, or both (Jansen 2020).

17 See, for example, Hurst 2008, Levine et al. 2004, Luna 2009, Nickel 2006, Schroeder and Gefenas 2009.

18 The general purpose of paragraph 29 can be read as ‘ensuring that patients are not disadvantaged or exploited when they take part in clinical trials’ (Lewis et al. 2002, p. 1337). The paragraph effectively proposes a ‘standard of care’ principle, a term that denotes the degree or quality of treatment adequate enough to fulfil the obligation the physician has to provide the best possible care for a patient. As the pharmaceutical market has grown, an increased amount of research has been conducted in low- or middle-income countries (LMICs), raising ethical concerns due to the disparities in education and healthcare between the host countries (or populations) and the countries where the research originates from and/or the results will be primarily used in (Glickman et al. 2009; Killen et al. 2002). The go-to example of this ethical tension is a number of trials conducted in the 1990s, in which antiretroviral drugs for maternal-fetal transmission of HIV were tested against placebo despite it being known at the time that zidovudine is effective at reducing the rate at which HIV-positive mothers transmit the virus to their offspring (Angell 1997; Lurie and Wolfe 1997; Wertheimer 2008). In this case, the trials were arguably designed to inform the practice of locals living in those (and similar) nations, in which zidovudine was not routinely available due to its cost (Wertheimer 2008). Thus, it might be argued that there was no ethical duty to use it as a reference when conducting a study in the population in question. Temple of the FDA (2007, p. 157), for example, asks ‘why, if everyone in a trial is better off because of participation, and no one is denied anything otherwise available to them, the trial is not ethically acceptable.’ Similarly, one might argue that nothing is truly lost when a drug that has been introduced for trial purposes is discontinued at the end of a trial. This type of argumentation, however, does not arguably cure our concerns about the potential unacceptability of certain trials. Some global oncology trials, for example, are clearly aimed at regulatory approval in the US and Europe, with the drug tested being far too expensive for use in the host country (Prasad et al. 2016). These types of trials, at least, raise ethical concerns regarding the potential exploitation of LMICs and populations/individuals in these countries (Macklin 2003). Even with assurances that participants fully understood the details of the trial and faced no clear pressure to consent, a persistent unease surrounds certain types of transactions that may be difficult to articulate within the framework of traditional liberal individualist bioethics (Malmqvist 2013; ten Have 2016). In addition to research off-shored to LMICs, such transactions include commercial surrogate motherhood, organ trading, and cancer patients hoping for a cure (ten Have 2016).

19 By incorporating any additional harms into its formulation – rather than only wrongful harms – the DoH subtly but significantly deviates from Hurst’s original definition. According to Hurst, a definition of vulnerability should not extend to all additional harms, as there is no duty to protect participants from every possible harm (Hurst 2008). Furthermore, it is noteworthy that the formulation in paragraph 19 does not strictly define particular vulnerability as an increased likelihood of being wronged or harmed. Instead, it leaves open the question of whether particular vulnerability always entails these risks.

20 The source of our obligations towards the vulnerable admittedly remains a point of contention in the literature on vulnerability. See for example Martin et al. (2014); Mackenzie (2016); Wrigley (2015).

21 The commentary on guideline 13, which concerns elderly persons, states this idea succinctly (CIOMS 2002, emphasis added): Elderly persons are commonly regarded as vulnerable. With advancing age, people are increasingly likely to acquire attributes that define them as vulnerable. They may, for example, be institutionalized or develop varying degrees of dementia. *If and when they acquire such vulnerability-defining attributes, and not before, it is appropriate to consider them vulnerable and to treat them accordingly.*
